# EBV Exploits RNA m^6^A Modification to Promote Cell Survival and Progeny Virus Production During Lytic Cycle

**DOI:** 10.3389/fmicb.2022.870816

**Published:** 2022-06-15

**Authors:** Yusuke Yanagi, Takahiro Watanabe, Yuya Hara, Yoshitaka Sato, Hiroshi Kimura, Takayuki Murata

**Affiliations:** ^1^Department of Virology, Nagoya University Graduate School of Medicine, Nagoya, Japan; ^2^Department of Virology and Parasitology, Fujita Health University School of Medicine, Toyoake, Japan

**Keywords:** EBV, lytic cycle, m^6^A modification, METTL3, 3-deazaadenosine

## Abstract

N6-methyladenosine (m^6^A) mediates various biological processes by affecting RNA stability, splicing, and translational efficiency. The roles of m^6^A modification in Epstein-Barr virus (EBV) infection in the lytic phase are unclear. Here, knockout of the m^6^A methyltransferase, N6-methyladenosine methyltransferase-like 3 (METTL3), or inhibition of methylation by DAA or UZH1a decreased the expression of viral lytic proteins and reduced progeny virion production. Interestingly, cell growth and viability were decreased by induction of the lytic cycle in METTL3-knockout or inhibitor-treated cells. Apoptosis was induced in those conditions possibly because of a decreased level of the anti-apoptotic viral protein, BHRF1. Therefore, m^6^A shows potential as a target of lytic induction therapy for EBV-associated cancers, including Burkitt lymphoma.

## Importance

The roles of m^6^A modification of EBV are unclear. Here, knockout of the methylation enzyme or pharmacological blockade of methylation showed that EBV exploits the m^6^A system for efficient lytic replication. Also, lytic induction and m^6^A inhibition synergistically decreased the viability of EBV-positive tumor cells, suggesting that m^6^A inhibitors, in conjunction with lytic induction, have therapeutic potential for EBV-associated diseases.

## Introduction

Epstein-Barr virus (EBV) is a ubiquitous human tumor virus in the gamma-herpesvirus subfamily (Cohen, 2000). Most children are asymptomatically infected with EBV, but first infection in and after adolescence frequently manifests infectious mononucleosis (Dunmire et al., 2015). EBV can also cause malignant disorders, including Burkitt lymphoma, Hodgkin lymphoma, nasopharyngeal carcinoma, gastric carcinoma, T/NK cell lymphoma, and chronic active EBV disease (Young et al., 2016).

EBV predominantly establishes latency in B cells, but is occasionally reactivated and enters the lytic cycle, leading eventually to progeny virion production (McKenzie and El-Guindy, 2015). In EBV-positive cancer cells, the virus is latent and expresses a few genes, such as EBV nuclear antigen (EBNA)-1, 2, 3, and leader protein, and latent membrane protein (LMP)-1 and 2 (Murata et al., 2014). These latent genes are involved in cell signal modification, cell growth promotion, prevention of cell death, and immune evasion.

Although the trigger for EBV reactivation *in vivo* is unclear, it can be induced *in vitro* by chemical, biological, or physical stimuli, such as 12-*O*-tetraadecanoylphorbol-13-acetate, sodium butyrate, transforming growth factor-β, anti-immunoglobulin (anti-Ig), hypoxia, and temperature shifting (Murata et al., 2021). Those stimuli induce the expression of two viral immediate-early (IE) genes, BZLF1 and BRLF1. Being transcriptional activators, these IE genes promote the transcription of early EBV genes, such as BALF5 (viral DNA polymerase catalytic subunit), BHRF1 (viral homolog of BCL2), and BMRF1 (viral DNA polymerase processivity factor), many of which mediate viral DNA synthesis. Late viral genes, typically encoding structural proteins, such as BKRF4 (a tegument protein), and BALF4 (glycoprotein B [gB]) are expressed next (Murata, 2018). Progeny virions are assembled in infected cells and released extracellularly.

N6-methyladenosine (m^6^A) modification is the most common type of modification of protein-coding mRNAs, transfer RNAs, ribosomal RNAs, long non-coding RNAs, microRNAs, and circular RNAs. The physiological roles of m^6^A modification are diverse; it has been implicated in splicing, decay, maturation, nuclear exportation, and translation initiation of RNA. m^6^A modification is thus implicated in biomolecule metabolism, circadian rhythm, development, regeneration, and medical disorders (Fu et al., 2014; Zaccara et al., 2019; Liu et al., 2020).

m^6^A modification also occur in viruses. For example, HIV RNA can undergo m^6^A modification, contributing to nuclear export of HIV RNA, which is packaged into capsids (Lichinchi et al., 2016). Hepatitis C virus (HCV) RNA bearing the m^6^A modification is packaged into capsids, whereas HCV RNA without m^6^A is retained in the cell (Gokhale et al., 2016). Kaposi sarcoma-associated herpesvirus (KSHV) uses the m^6^A modification to promote lytic replication (Ye et al., 2017), but this is cell-type dependent (Hesser et al., 2018; Tan et al., 2018). m^6^A modification of latent and lytic EBV mRNAs increased the stability of latent gene mRNAs, whereas that of lytic genes promoted the degradation of their transcripts, suggesting that methylation contributes to the maintenance of EBV latency (Lang et al., 2019; Zheng et al., 2021). Moreover, m^6^A modification of some EBV lytic genes mediated RNA decay, resulting in decreased viral replication (Xia et al., 2021; Zhang et al., 2021). Expression of the methylation enzyme is reportedly inversely correlated with that of BZLF1 (Dai et al., 2021).

We examined the role of m^6^A modification in the lytic cycle of EBV using knockout (KO) technology and a pharmacological inhibitor. Inhibition of m^6^A decreased the production of viral lytic proteins and progeny virions. Moreover, simultaneous lytic induction and inhibition of m^6^A modification markedly suppressed the viability and proliferation of EBV-positive cancer cells, suggesting m^6^A modification to be a potential target for lytic induction therapy.

## Materials and Methods

### Cells and Reagents

EBV-positive and -negative HEK293 cells were cultured in Dulbecco's modified Eagle's medium supplemented with 10% fetal bovine serum (FBS). EBV-positive and -negative Akata (Shimizu et al., 1994) and AGS/EGFP-EBV (Katsumura et al., 2009) cells were cultured in Roswell Park Memorial Institute medium supplemented with 10% FBS. BZLF1 expression plasmids were prepared as previously described (Murata et al., 2009). Construction of the N6-methyladenosine methyltransferase-like 3 (METTL3)-KO vector was mediated by the CRISPR/Cas9 system. The following sequences were inserted into the PX459 vector (Addgene): sense 5′-CACCGAGGCAGCATTGTCTCCAACG-3′ and antisense 5′-AAACGTTGGAGACAATGCTGCCTC-3′. Previously reported antibodies against LMP1, EBNA1, BZLF1, BMRF1, BKRF4, and BALF4 were used (Masud et al., 2017; Yanagi et al., 2021). Antibodies against BHRF1, METTL3, and GAPDH were purchased from Merck Millipore, Abcam, and Cell Signaling, respectively. Horseradish peroxidase-conjugated secondary mouse and rabbit IgGs were purchased from Amersham Biosciences. 3-Deazaadenosine (DAA) and UZH1a were obtained from Sigma and MedChemExpress, respectively.

### Preparation of EBV-Positive METTL3-KO Cell Lines

To knock out the METTL3 gene, EBV-negative HEK293 cells were transfected with the METTL3-KO plasmid vector using Lipofectamine 2000 and cloned in the presence of 1 μg/mL puromycin. Disruption of the gene was confirmed by sequencing and immunoblotting. To obtain EBV-positive HEK293 cell lines, METTL3-KO and wild-type (control) HEK293 cell clones were transfected with B95-8 strain EBV bacterial artificial chromosome (BAC) with Lipofectamine 2000, and then infected cells were selected with 150 μg/mL hygromycin.

To produce METTL3-KO B cells, EBV-negative Akata cells were transfected with the METTL3-KO vector by electroporation at 1,150 V with two 20-ms pulses using the Neon Transfection System (Thermo Fisher Scientific). After puromycin (1 μg/mL) selection for 2 days, limited dilution was conducted to obtain cell clones. Disruption of the gene was confirmed by sequencing and immunoblotting. Wild-type and KO cells were infected with Akata-strain EBV produced in AGS/EGFP-EBV cells, and infected cells were selected using 750 μg/ml G418.

### Induction of the Lytic Cycle

EBV-positive HEK293 cells were transfected with BZLF1 expression plasmid by electroporation at 1,100 V with three 10-ms pulses using the Neon Transfection System (Thermo Fisher Scientific). EBV-positive Akata cells were treated with an anti-IgG antibody.

### Immunoblotting

Immunoblotting was carried out as described previously (Yanagi et al., 2019). Cellular protein was harvested *via* sonication in sample buffer containing sodium dodecyl sulfate (SDS) and 2-mercaptoethanol and subjected to SDS-polyacrylamide electrophoresis (PAGE). The proteins were transferred to a polyvinylidene fluoride membrane, followed by antibody treatment and visualization of chemiluminescence.

### Quantification of RNA

For reverse transcriptase-quantitative polymerase chain reaction (RT-qPCR), total RNAs were extracted and quantified using the One-Step TB Green PrimeScript™ Plus RT-PCR Kit (TaKaRa Bio), as described previously (Yanagi et al., 2021). The following primer sequences were used: GAPDH, 5′-TGCACCACCAACTGCTAGC-3′ and 5′-GGCATGGACTGTGGTCATGAG-3′; EBNA1, 5′- GGCTAGGAGTCACGTAGA-3′ and 5′- GGAACAGCAAGGGCAATT-3′; EBNA2, 5′-TTAGAGAGTGGCTGCTACGCATT-3′ and 5′-TCACAAATCACCTGGCTAAG-3′; LMP1, 5′-GATGATCACCCTCCTACT-3′ and 5′-GCCAGAGAATCTCCAAGA-3′; BZLF1, 5′-CAGAATCGCTGGAGGAAT-3′ and 5′-AAGCCACCTCACGGTAGT-3′; BHRF1, 5′-TGGCCTATTCAACAAGGGAG-3′ and 5′-TTTCTCTTGCTGCTAGCTCC-3′; BMRF1, 5′-CAACACCGCACTGGAGAG-3′ and 5′-GCCTGCTTCACTTTCTTGG-3′; BKRF4, 5′-GTGGCATCCTTCAGATTC-3′ and 5′-CCTGAAGACTGTTGGGTA-3′; BALF4, 5′-GAGTACAACTTCCAGGCG-3′ and 5′-GCTACCCAGACTGTCCAT-3′.

### Quantification of Viral DNA Replication

The viral DNA level in cells was measured as described previously (Yanagi et al., 2019). Briefly, after washing with phosphate-buffered saline, cells were lysed *via* sonication and treated with proteinase K. Quantitative PCR was performed using Fast Start Universal Probe Master Mix (Roche).

### Quantification of Progeny Virus Infectivity

EBV titers were measured based on green fluorescent protein (GFP) positivity because both EBVs encode enhanced GFP (EGFP). Cell culture supernatants were collected and inoculated with 1 × 10^6^ EBV-negative Akata cells. After 2 days, EGFP positivity was determined using fluorescence-activated cell sorting with a FACS Calibur G5 instrument (Becton Dickinson).

### MeRIP Assay

Lytic reactivation of EBV was induced by BZLF1 transfection or anti-IgG treatment of EBV-positive HEK293 or EBV-positive Akata cells, respectively, for 2 days. Total RNA was harvested using the RNeasy Mini Kit (Qiagen) and treated using the Magna MeRIP m6A Kit (Merck). RNA was quantified *via* RT-qPCR using primers specific for a m^6^A-modified site in the BMRF1 gene (Xia et al., 2021): 5′-CTCTCCGCTTTAAGACCAAG-3′ and 5′-AGGTTTACCTGCAGCACTCC-3′.

### Cell Growth and Viability

The number of live cells was counted on days 0, 2, 4, 6, and 8. Cells were stained with trypan blue using Countess II FL (Invitrogen). Cell growth was defined as the ratio of the number of live cells to that on day 0. Live and total cells were counted on days 0, 2, 4, 6, and 8. Cell viability was defined as the ratio of the number of live cells to that of total cells.

## Results

### Importance of METTL3 for Efficient Production of EBV Progeny

**Figure 1:**
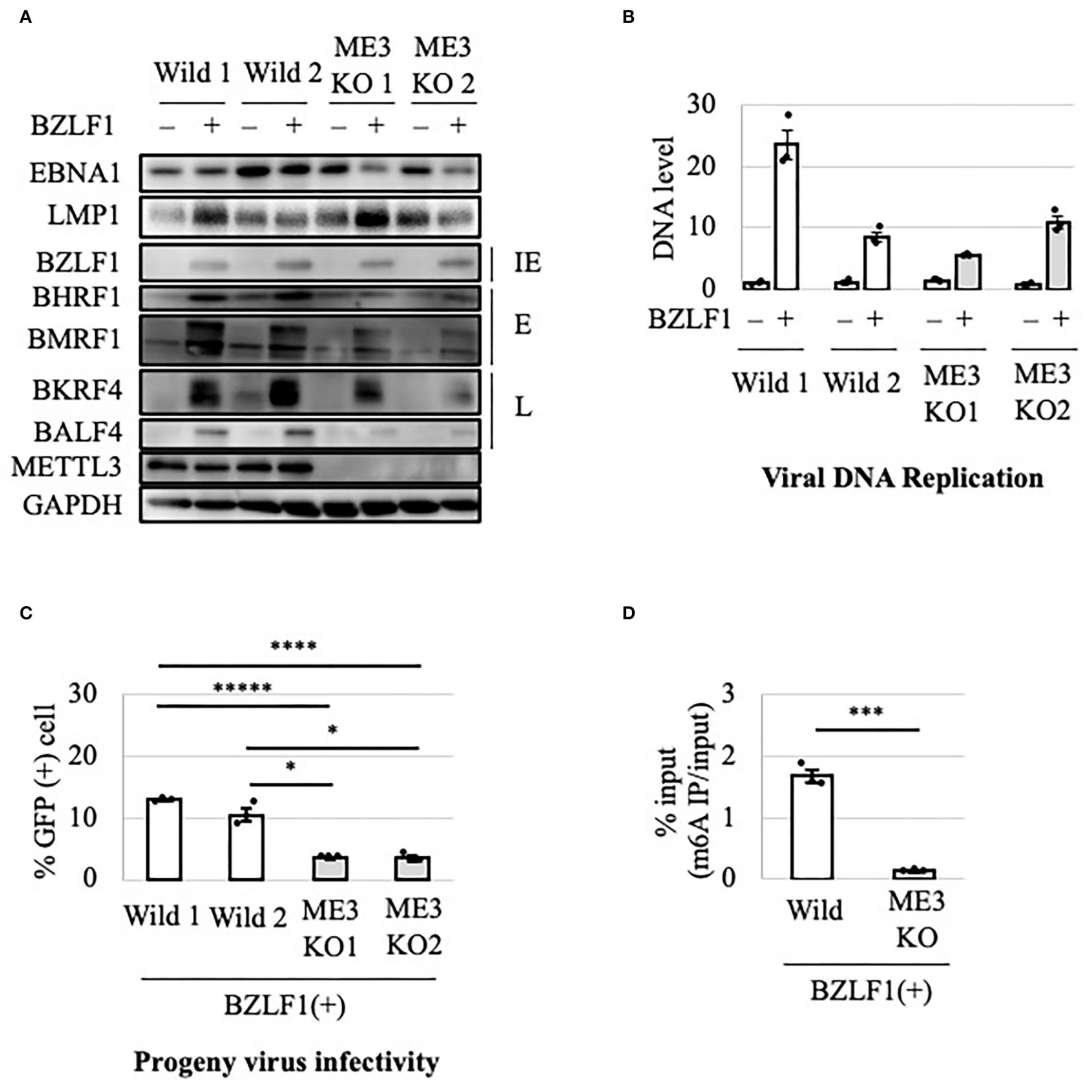
Importance of METTL3 for efficient production of EBV progeny in HEK293 cells. **(A)** Epstein-Barr virus (EBV)-positive wild-type (Wild) and METTL3-KO (ME3KO) HEK293 cells were transfected with BZLF1 expression plasmids to induce the lytic cycle. Cell proteins were collected before lytic induction [BZLF1(–)] or at 2 days after BZLF1 transfection [BZLF1(+)] and subjected to immunoblotting. IE, E, and L denote immediate-early, early, and late genes, respectively. **(B)** Cell DNA was extracted before lytic induction [BZLF1(–)] or at 2 days after BZLF1 transfection [BZLF1(+)], and EBV DNA was quantified using qPCR. The means ± SDs of three biological replicates normalized to the host DNA level are presented. **(C)** Lytic induction was carried out as in **(A)**. Culture media were collected on day 3 after transfection, and supernatants were inoculated with EBV-negative Akata cells. The percentage of infected cells was measured based on GFP positivity using fluorescence-activated cell sorting (FACS). The means ± SDs of three biological replicates are presented. **(D)** Cells were transfected with the BZLF1 expression vector to induce the lytic cycle. At 2 days after transfection, cell RNA was collected and subjected to MeRIP-PCR. Methylation levels are shown as percentages of input. *p < 0.05, ***p < 0.005, ****p < 0.001, and *****p < 0.0005.

To evaluate the role of m^6^A in the EBV lytic cycle, METTL3 was knocked out in EBV-negative HEK293 cells using the CRISPR/Cas9 system. After confirmation of METTL3 KO, the cell clones were transfected with EBV-BAC DNA in parallel with control cells (wild type), followed by hygromycin selection to obtain EBV-positive cells (Wild1, Wild2, ME3KO1, and ME3KO2). Reactivation was induced by transfection of the BZLF1 expression vector and cell protein was harvested on day 0 or 2 after infection. METTL3 protein was not detected *via* immunoblotting in the KO cells (Figure 1A). BZLF1 protein levels were similar, and those of early and late gene products were decreased in the KO cells (Figure 1A). Viral DNA synthesis took place after 2 days in both wild-type and KO cells (Figure 1B). KO of METTL3 had a marginal effect on viral DNA replication. Wild1 supported viral replication more efficiently than the other cells, albeit within the range of fluctuation. Production of infectious progeny virions by KO cells was significantly lower compared to wild-type cells (Figure 1C). This was likely caused by lower levels of viral proteins needed for efficient progeny production, such as BALF4 (gB) (Figure 1A). Also, the m^6^A modification level was low in the KO cells (Figure 1D). Similar experiments using samples harvested at later time points (days 3 and 4) (Supplementary Figure 1) yielded similar results.

**Figure 2:**
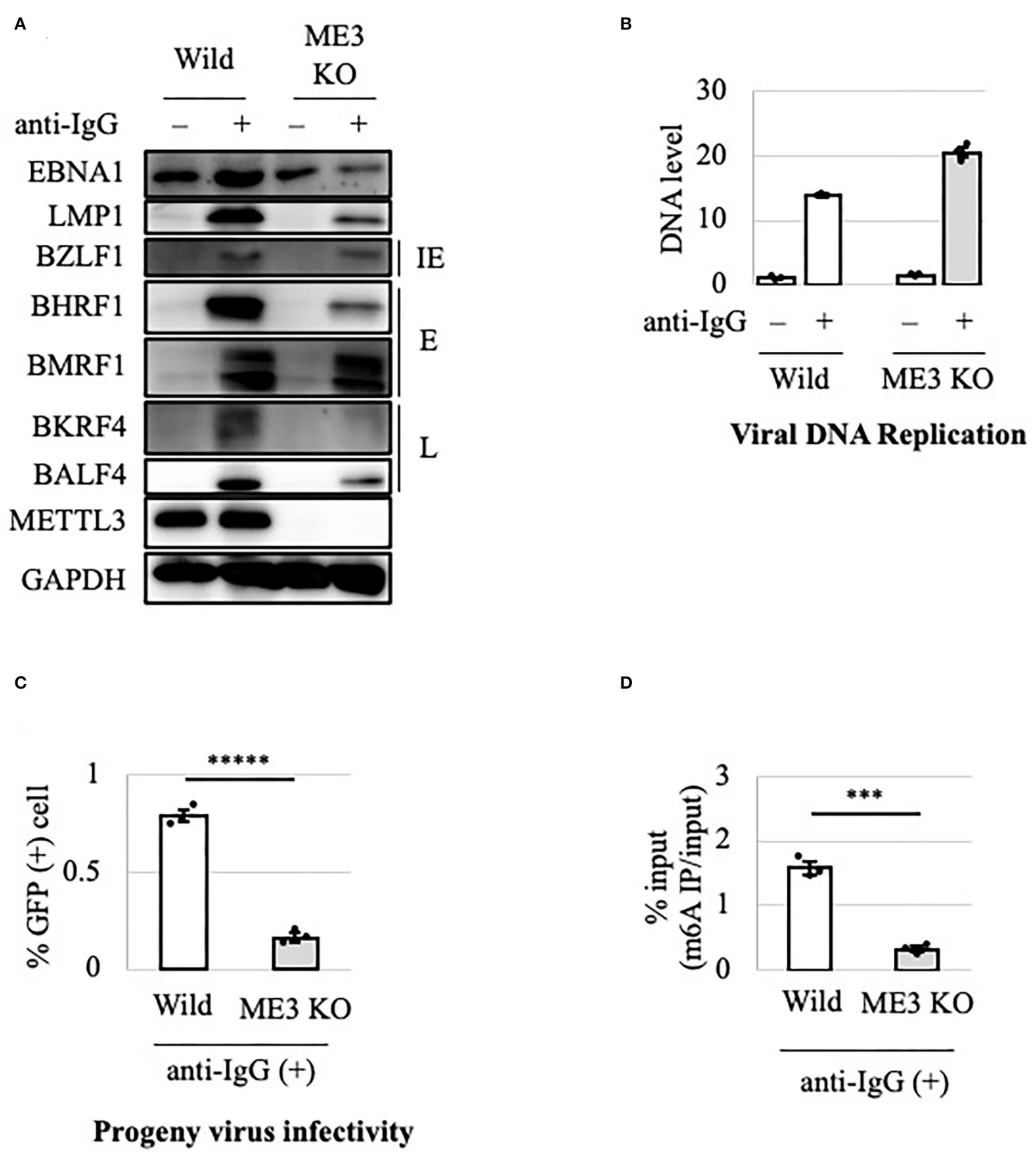
Importance of METTL3 for efficient production of EBV progeny in Akata cells. **(A)** EBV-positive wild-type (Wild) and METTL3-KO (ME3KO) Akata cells were treated with anti-IgG to induce the lytic cycle. Cell proteins were collected before lytic induction [anti-IgG(–)] or at 2 days after anti-IgG addition [anti-IgG(+)] and subjected to immunoblotting. IE, E, and L denote immediate-early, early, and late genes, respectively. **(B)** Cell DNA was extracted before lytic induction [anti-IgG(–)] or at 2 days after anti-IgG addition [anti-IgG(+)], and EBV DNA was quantified using qPCR. The means ± SDs of three biological replicates normalized to the host DNA level are presented. **(C)** Lytic induction was carried out as in **(A)**. Culture media were collected on day 3, and supernatants were inoculated with EBV-negative Akata cells. The percentage of infected cells was measured based on GFP positivity using FACS. The means ± SDs of three biological replicates are presented. **(D)** Cells were treated with anti-IgG to induce the lytic cycle. At 2 days after treatment, cell RNA was collected and subjected to MeRIP-PCR. Methylation levels are shown as percentages of input. ***p < 0.005 and *****p < 0.0005.

Because HEK293 is not a natural host cell type for EBV, we examined the effect of METTL3 KO in B cells. The METTL3 gene was knocked out using CRISPR/Cas9 in EBV-negative Akata cells. The KO and control (wild-type) cell clones were infected with EBV produced by AGS/EGFP-EBV cells. Because the EBV genome encodes a G418-resistance gene, the cells were treated with G418 to obtain EBV-positive cells. The lytic cycle was induced by the addition of anti-IgG and the cells were incubated for 0 or 2 days. As in HEK293-based cells, production of viral early and late proteins was decreased in METTL3-KO cells (Figure 2A). METTL3 protein was absent in METTL3-KO cells. The viral DNA level in METTL3-KO cells after lytic induction for 2 days was similar to that in wild-type cells (Figure 2B). Progeny production in Akata cells was significantly suppressed by METTL3 KO (Figure 2C). The m^6^A modification level was significantly low in the KO cells (Figure 2D). Similar results were obtained using samples harvested at different time points (Supplementary Figure 2). Therefore, m^6^A is important for efficient execution of the EBV lytic cycle.

### Viral Lytic mRNAs Were Differentially Regulated by METTL3

**Figure 3:**
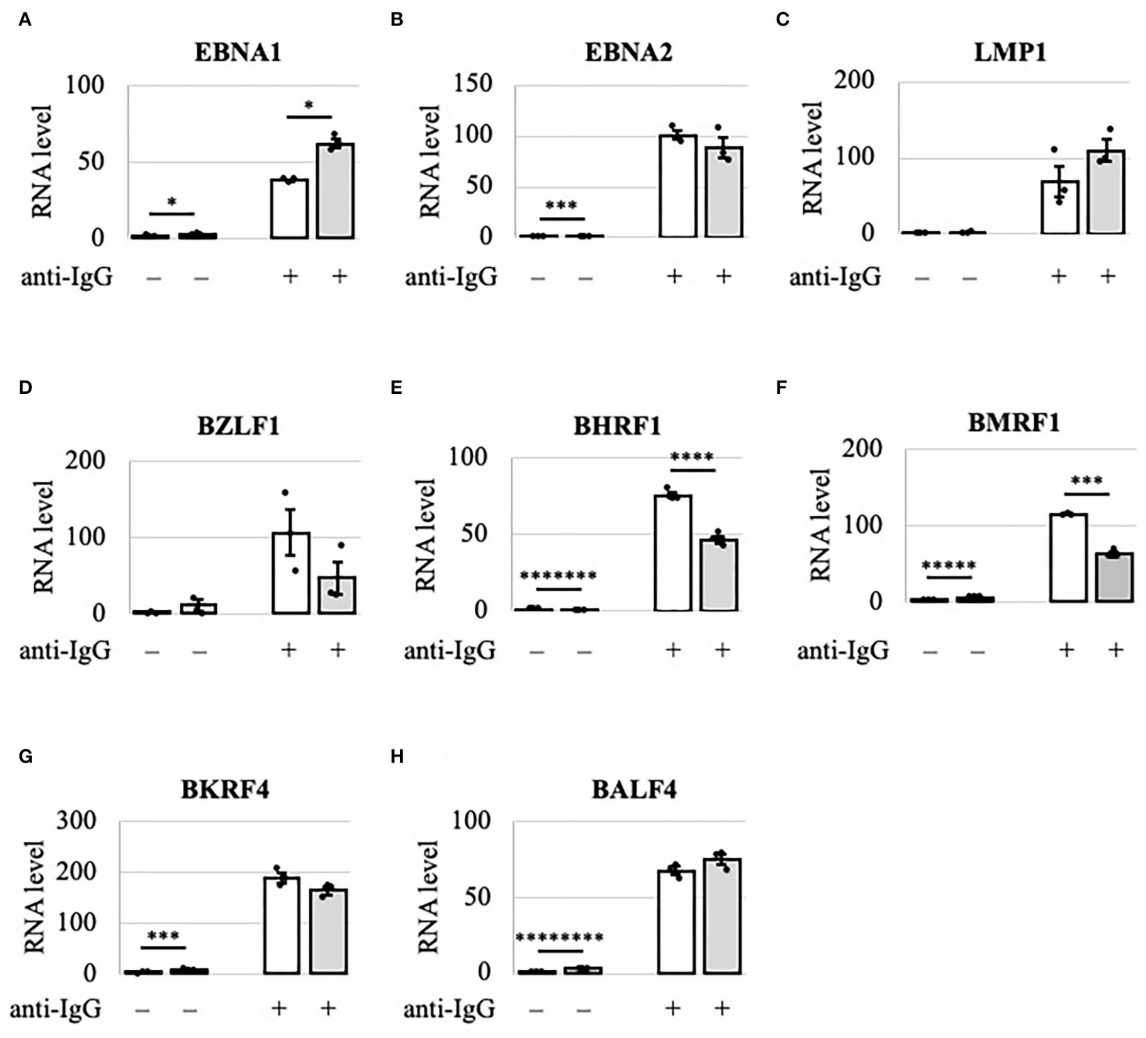
Viral lytic mRNAs were differentially regulated by METTL3 in Akata cells. EBV-positive wild-type (white bars) and METTL3-KO (gray bars) Akata cells were treated with anti-IgG to induce the lytic cycle. RNA was extracted from uninduced cells [anti-IgG(–)] or cells treated with anti-IgG for 2 days [anti-IgG(+)] and subjected to RT-qPCR. **(A)** EBNA1, **(B)** EBNA2, **(C)** LMP1, **(D)** BZLF1, **(E)** BHRF1, **(F)** BMRF1, **(G)** BKRF4, and **(H)** BALF4. The means ± SDs of three biological replicates normalized to the host GAPDH level are presented. *p < 0.05, ***p < 0.005, ****p < 0.001, *****p < 0.0005, *******p < 0.00005, and ********p < 0.00001.

Because viral lytic protein expression was decreased by METTL3 KO, we examined using RT-qPCR the viral mRNA levels in Akata cells after lytic induction by anti-IgG (Figure 3). IE and early gene transcripts (BZLF1, BHRF1, and BMRF1) were decreased by KO on day 2 (Figures 3D–F, gray bars), consistent with the decreased protein expression on day 2 (Figure 2A). Interestingly, although the levels of viral late lytic proteins (BKRF4 and BALF4) were reduced by METTL3 KO in Akata cells (Figure 2A), the mRNA levels of BKRF4 and BALF4 were not significantly decreased post-KO on day 2 (Figures 3G,H). Therefore, IE and early genes are regulated differently by m^6^A modification vs. late genes; IE/early transcripts may be stabilized by m^6^A, whereas m^6^A modification of late-gene mRNAs may trigger efficient translation.

### METTL3 Is Required for EBV-Positive Cell Viability in the Lytic Cycle

**Figure 4:**
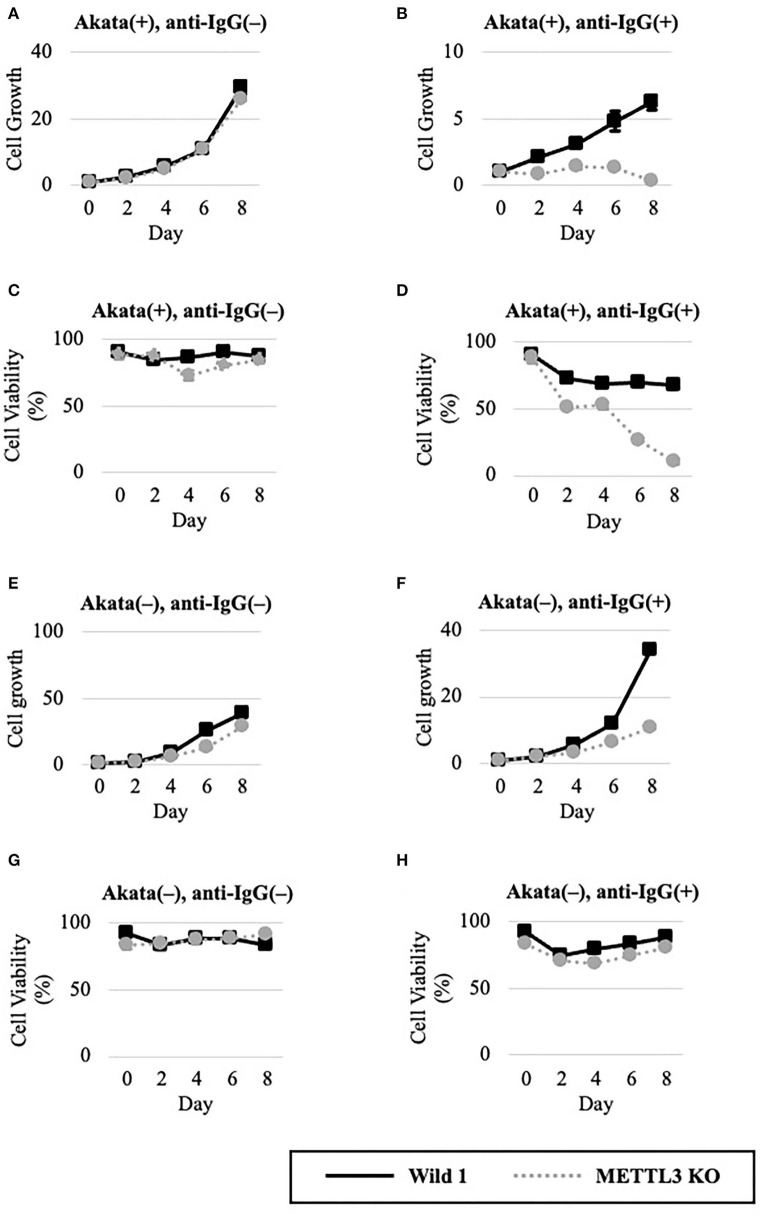
METTL3 was required for the efficient growth of EBV-positive Akata cells in the lytic cycle. EBV-positive **(A–D)** or EBV-negative **(E–G)**, wild-type (Wild, black lines), and METTL3-KO (dashed gray lines) Akata cells were seeded and cultured in the presence **(B,D,F,H)** or absence **(A,C,E,G)** of anti-IgG. On the indicated days after induction, cells were stained with trypan blue, and trypan blue-positive and -negative cells were counted. Cell growth **(A,B,E,F)** was calculated as the ratio of the number of live (trypan blue-negative) cells to that on day 0. **(C,D,G,H)** Ratio of the number of live cells to the total number of cells. The means ± SDs of two biological replicates are presented.

We next evaluated METTL3 as a molecular target for anticancer drug development. To this end, wild-type and KO clones of EBV-positive Akata cells were cultured in the presence or absence of lytic induction (Figures 4A–D). Without anti-IgG, cell growth and viability were unchanged (Figures 4A,C). Interestingly, however, the growth and viability of EBV-positive Akata-KO cells were decreased by anti-IgG (Figures 4B,D), suggesting that METTL3 prevents cell death during the EBV lytic cycle. One may argue that EBV reactivation might not be efficiently executed in this experiment and that the observed effect by the METTL3KO and anti-IgG might be accounted by cells in which viral reactivation did not take place. However, Figures 4A,C show that cell growth and viability of EBV-positive Akata cells that did not undergo lytic reactivation were not affected by the KO.

To determine whether EBV is needed for that process, we carried out similar experiments in EBV-negative Akata cells (Figures 4E–H). Without lytic induction, growth and viability were not affected by METTL3 KO (Figures 4E,G). In the presence of anti-IgG, METTL3 KO had decelerated growth on days 6 and 8 after induction, albeit not zero (Figure 4F). Cell viability was decreased only slightly (Figure 4H). Therefore, METTL3 is important for the growth and survival of EBV-positive cancer cells during the EBV lytic cycle.

### Effectiveness of m^6^A Pharmacological Inhibition

**Figure 5:**
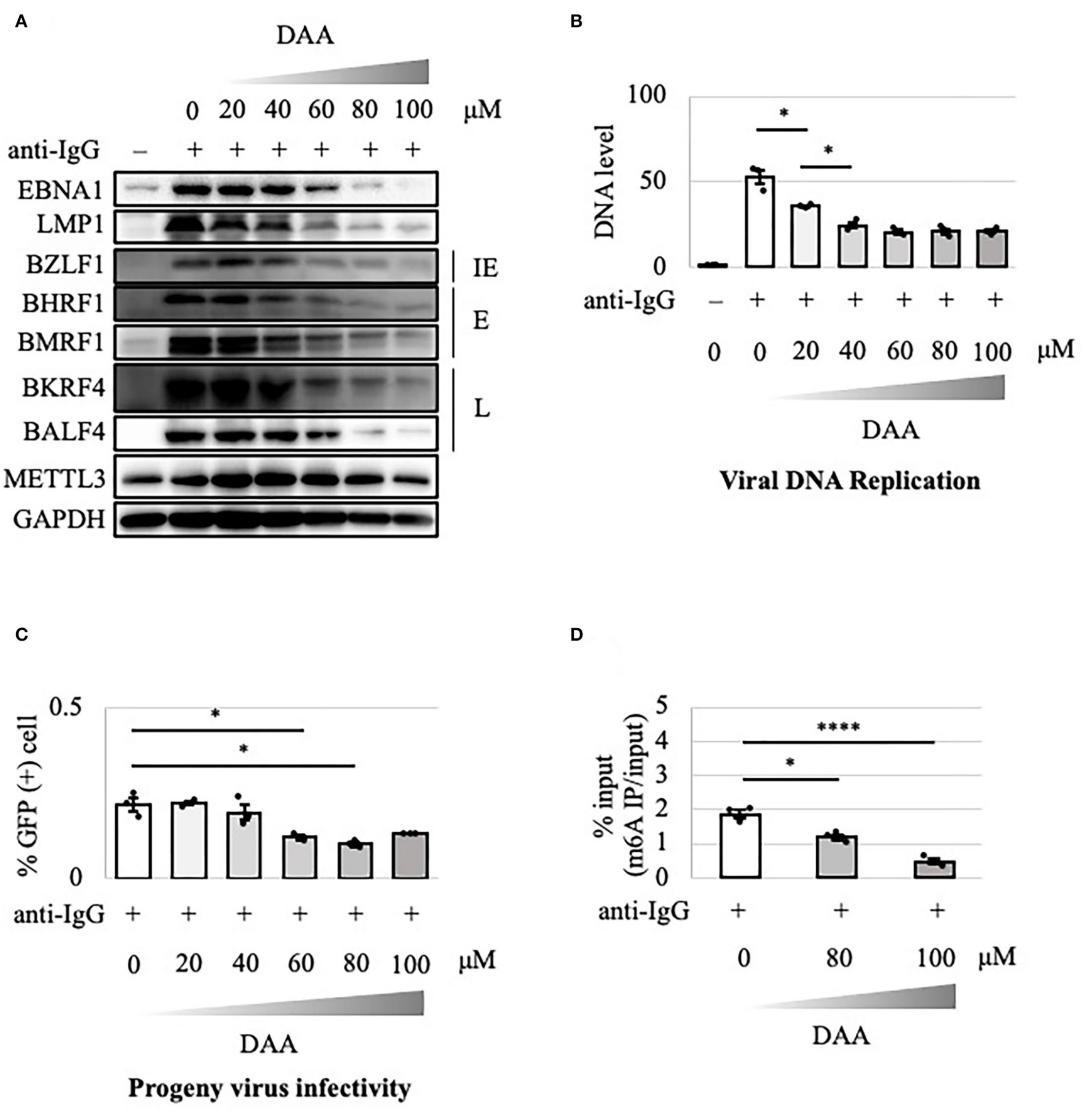
Pharmacological inhibition of m^6^A by DAA suppressed lytic replication in Akata cells. **(A)** EBV-positive wild-type Akata cells were treated or not treated with anti-IgG and the indicated concentrations of DAA. Proteins were collected from uninduced cells [anti-IgG(–)] and cells treated with [anti-IgG(+)] and DAA for 2 days and subjected to immunoblotting. IE, E, and L denote immediate-early, early, and late genes, respectively. **(B)** Cell DNA was extracted before lytic induction [anti-IgG(–)] or at 2 days after [anti-IgG(+)] and DAA addition, and EBV DNA was quantified using qPCR. The means ± SDs of three biological replicates normalized to the host DNA level are presented. **(C)** Lytic induction and DAA treatment were carried out as in **(A)**. Culture media were collected on day 3, and supernatants were inoculated with EBV-negative Akata cells. The percentage of infected cells was measured based on GFP positivity using FACS. The means ± SDs of three biological replicates are presented. **(D)** Cells were treated with anti-IgG and DAA. On day 2 after treatment, cell RNA was collected and subjected to MeRIP-PCR. Methylation levels are shown as the percentages of input *p < 0.05 and ****p < 0.001.

The above results suggest that the METTL3 gene is a potential target in anticancer drug development. DAA inhibits m^6^A methylation by blocking the incorporation of S-adenosylmethionine. EBV-positive Akata cells were treated with anti-IgG and various concentrations of DAA for 2 days. Levels of latent and lytic viral proteins were decreased by DAA in a dose-dependent manner, whereas METTL3 and GAPDH were unaffected (Figure 5A). Accordingly, viral DNA synthesis and progeny production were significantly reduced by DAA (Figures 5B,C). Moreover, the m^6^A level was decreased by DAA in a dose-dependent manner (Figure 5D).

**Figure 6:**
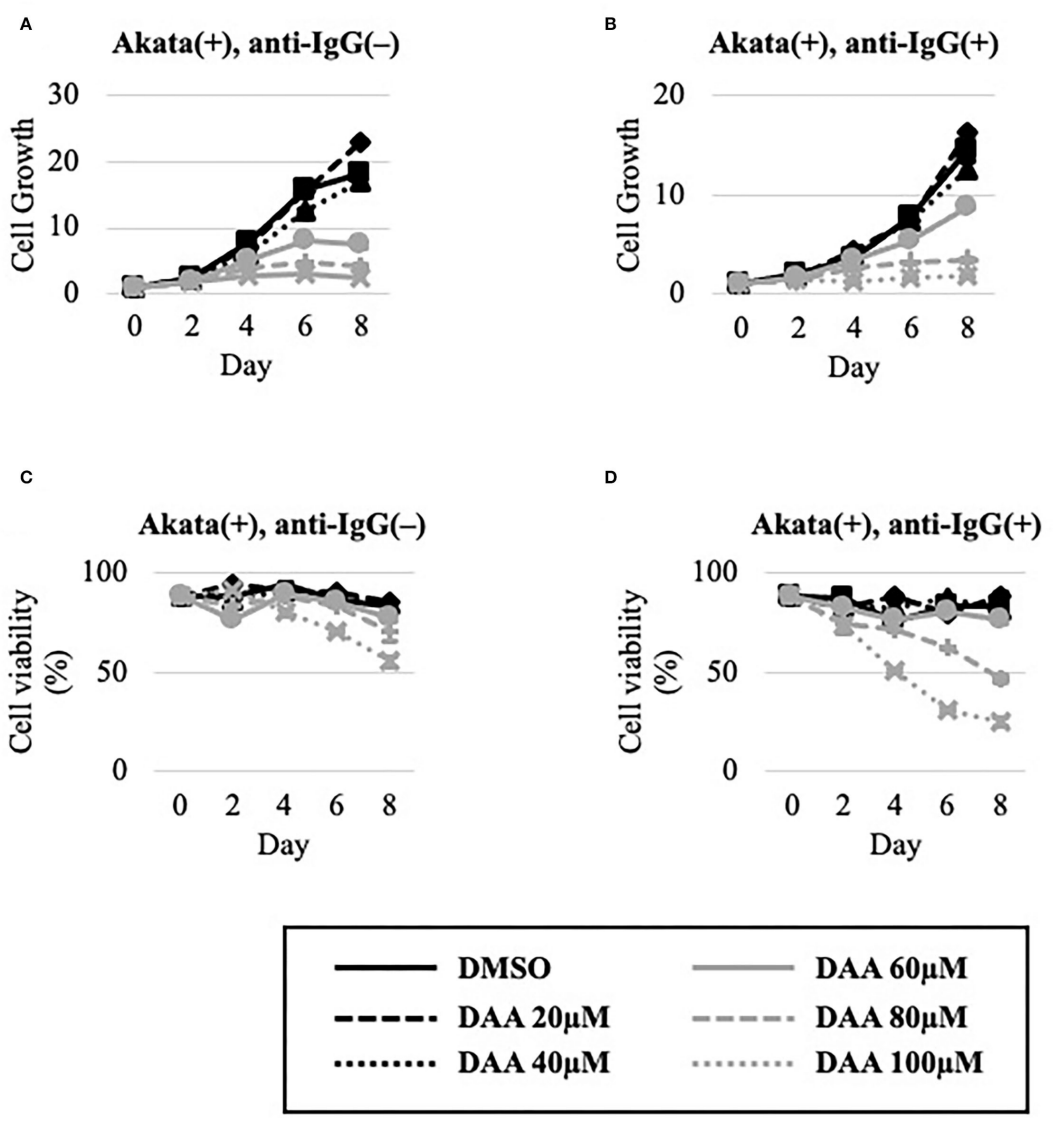
DAA decreased the growth and viability of Akata cells. EBV-positive Akata cells were seeded and cultured in the presence **(B,D)** or absence **(A,C)** of anti-IgG. Cells were simultaneously treated with the vehicle [dimethyl sulfoxide (DMSO)] or DAA at the indicated concentrations. On days 0, 2, 4, 6, and 8 after induction, cells were stained with trypan blue, and trypan blue-positive and -negative cells were counted. Cell growth **(A,B)** was calculated as the ratio of the number of live (trypan blue-negative) cells to that on day 0. **(C,D)** Ratio of the number of live cells to the total number of cells. The means ± SDs of two biological replicates are presented.

The effect of DAA on the growth and viability of EBV-positive Akata cells in the absence or presence of lytic induction by anti-IgG was examined (Figure 6). Unlike METTL3-KO cells (Figures 4A,C), DAA potently decreased cell growth and viability even in the latent state (Figures 6A,C). Lytic induction further decreased cell growth and viability in a dose-dependent manner (Figures 6B,D).

**Figure 7:**
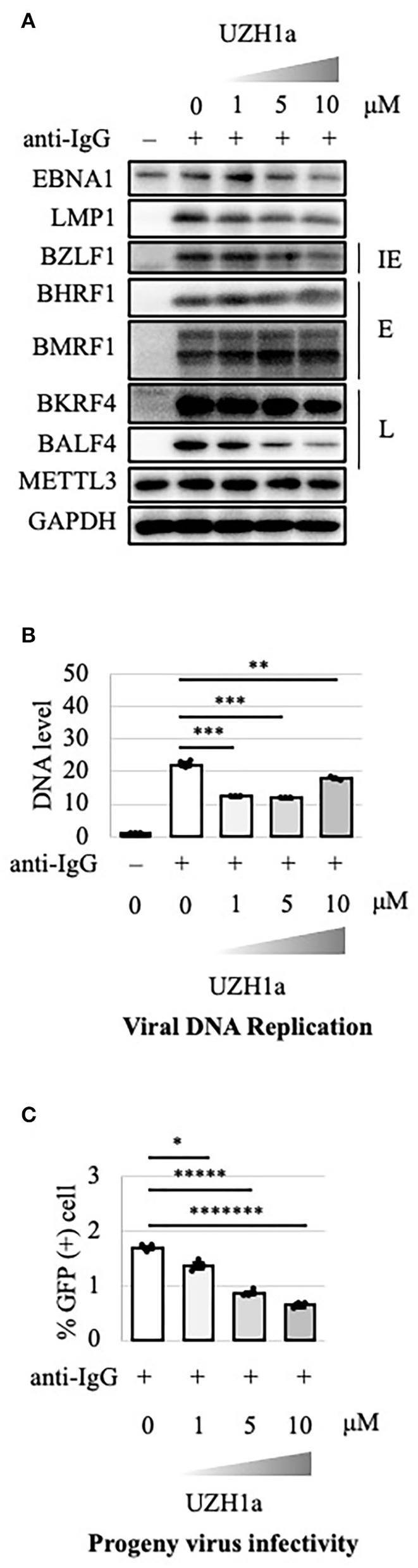
Pharmacological inhibition of m^6^A by UZH1a decreased lytic replication in Akata cells. **(A)** EBV-positive wild-type Akata cells were treated or not treated with anti-IgG and the indicated concentrations of UZH1a. Proteins were collected from uninduced cells [anti-IgG(–)] and cells treated with [anti-IgG(+)] and UZH1a for 2 days and subjected to immunoblotting. IE, E, and L denote immediate-early, early, and late genes, respectively. **(B)** DNA was extracted before lytic induction [anti-IgG(–)] or at 2 days after [anti-IgG(+)] and UZH1a addition, and EBV DNA was quantified using qPCR. The means ± SDs of three biological replicates normalized to the host DNA level are presented. **(C)** Lytic induction and UZH1a treatment were carried out as in **(A)**. Culture media were collected on day 3, and supernatants were inoculated with EBV-negative Akata cells. The percentage of infected cells was measured based on GFP positivity using FACS. The means ± SDs of three biological replicates are presented. *p < 0.05, **p < 0.01, ***p < 0.005, *****p < 0.0005, and *******p < 0.00005.

**Figure 8:**
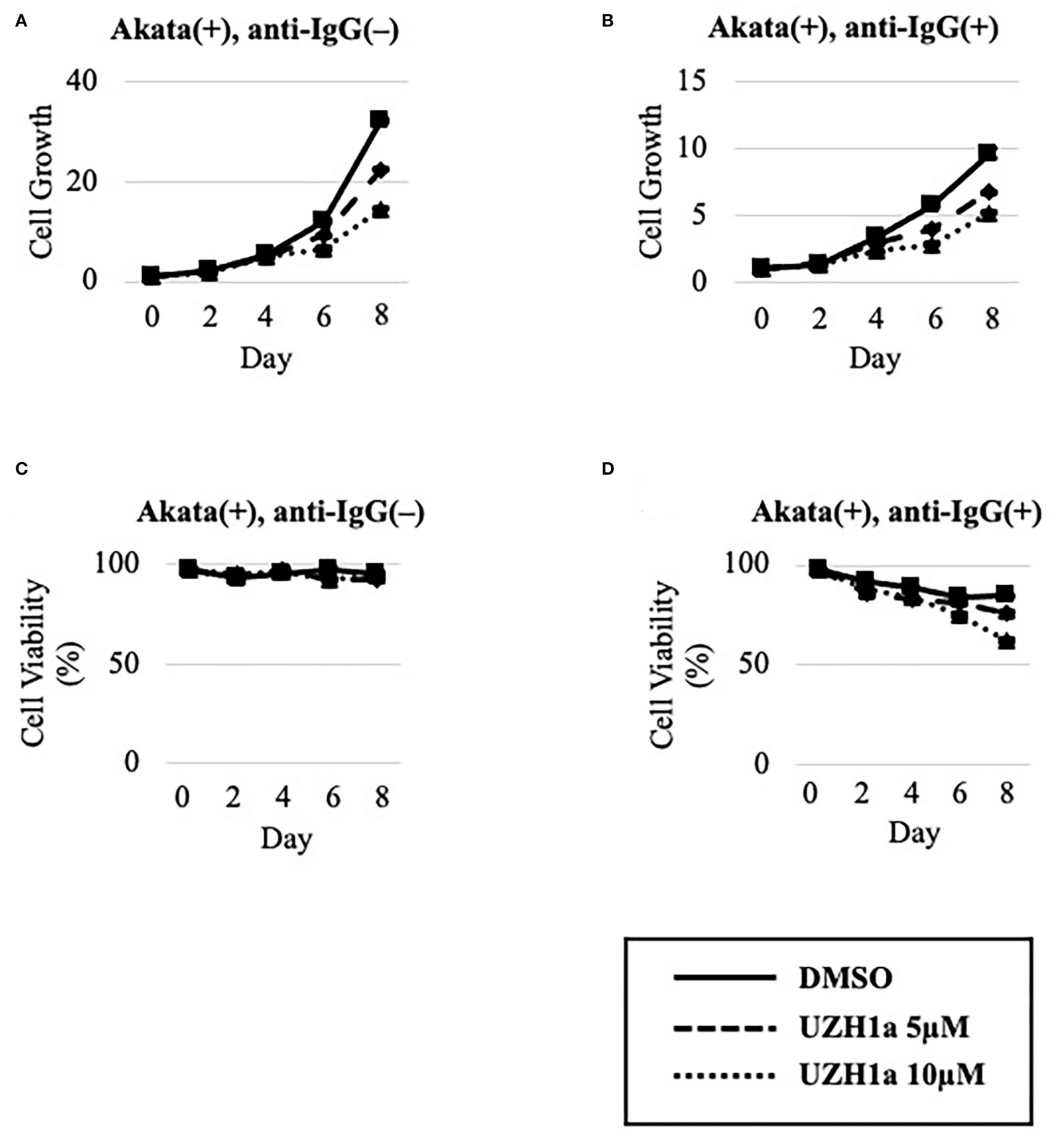
UZH1a decreased the growth and viability of Akata cells. EBV-positive Akata cells were seeded and cultured in the presence **(B,D)** or absence **(A,C)** of anti-IgG. Cells were simultaneously treated with DMSO (vehicle) or UZH1a at the indicated concentrations. On days 0, 2, 4, 6, and 8 after induction, cells were stained with trypan blue, and the numbers of trypan blue-positive and -negative cells were counted. Cell growth **(A,B)** was calculated as the ratio of the number of live (trypan blue-negative) cells to that on day 0. **(C,D)** Ratio of the number of live cells to the total number of cells. The means ± SDs of two biological replicates are presented.

To extend the results, we tested the effect of a specific inhibitor of METTL3, UZH1a. In the lytic state, the expression of some viral genes such as BKRF4 and BALF4 was decreased by UZH1a in a dose-dependent manner (Figure 7A). UZH1a also decreased viral DNA replication (Figure 7B) and progeny production (Figure 7C) as well as the growth and viability of EBV-positive Akata cells (Figure 8).

These results, combined with the data in Figures 1, 2, suggest that m^6^A modification of RNA plays an important role in EBV lytic replication and cell survival.

### Induction of Apoptosis in the Lytic Cycle

**Figure 9:**
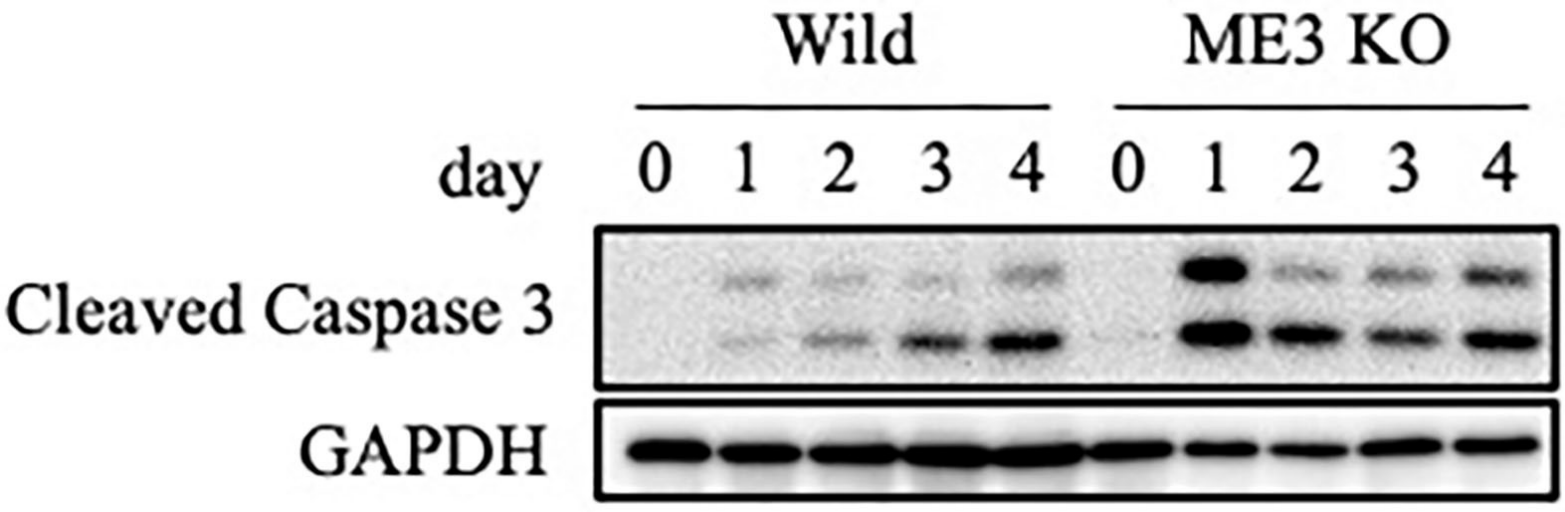
Expression of an apoptosis marker, cleaved caspase-3. EBV-positive wild-type Akata cells were treated with anti-IgG. Proteins were collected on days 0, 1, 2, 3, and 4 after induction and subjected to immunoblotting.

We evaluated the mechanism underlying the suppression by m^6^A inhibition of cell growth and viability in the lytic cycle. Wild-type or METTL3-KO EBV-positive Akata cells were treated with anti-IgG, and proteins were harvested for immunoblotting. The level of cleaved caspase-3, the activated form of a key factor in apoptosis, was increased by anti-IgG in both wild-type and METTL3-KO cells, compared to uninduced sample (day 0); the increase was greater in the latter, particularly on days 1 and 2 (Figure 9). Therefore, apoptosis is induced during the lytic cycle and suppressed by METTL3. We speculate that the lower expression of BHRF1 in KO cells (Figure 2A) mediated the induction of apoptosis.

## Discussion

m^6^A modification is a type of post-transcriptional modification conserved in many species, including yeast, plants, and mammals, and is involved in many biological processes. The RNAs of viruses—such as HIV, HCV, influenza A virus, respiratory syncytial virus, vesicular stomatitis virus, enterovirus 71, zika virus, dengue virus, severe acute respiratory syndrome coronavirus 2, hepatitis B virus, KSHV, human cytomegalovirus, herpes simplex virus, and adenovirus—also undergo such methylation (Baquero-Perez et al., 2021; Yu et al., 2021). However, the effect of m^6^A modification on viral replication varies according to the virus species and cell type. For instance, m^6^A modification was found to both increase and decrease KSHV replication, depending on the conditions (Ye et al., 2017; Hesser et al., 2018; Tan et al., 2018). The biological role of m^6^A modification in EBV lytic replication was still unclear (Lang et al., 2019; Dai et al., 2021; Xia et al., 2021; Zhang et al., 2021; Zheng et al., 2021). To provide insight into this issue, we prepared METTL3-KO HEK293 and Akata cell lines in which a crucial component of the methylation enzyme is knocked out in cells. KO is superior to knockdown (KD) because KD does not prevent expression over a long period due to the presence of unsilenced residual protein. In HEK293 and Akata cells, KO of METTL3 decreased the expression of viral lytic proteins and thereby suppressed progeny virion production (Figures 1, 2). Inhibitors of methylation, DAA and UZH1a, suppressed lytic viral protein expression and progeny virus production (Figures 5, 7). Therefore, m^6^A modification plays a supportive role in EBV lytic replication.

The viability and growth of EBV-positive Burkitt lymphoma Akata cells were suppressed by simultaneous anti-IgG treatment and m^6^A modification blockade, either by KO of METTL3 (Figure 4) or by inhibitors of m^6^A modification (Figures 6, 8). The reduction in the level of the viral anti-apoptotic protein, BHRF1, by METTL3 KO (Figures 1A, 2A) and pharmacological inhibition (Figures 5A, 7A) indicates induction of apoptosis. These data point toward a new combination therapy for Burkitt lymphoma, a highly aggressive form of B-cell lymphoma that grows very rapidly and has a poor prognosis because of chemotherapy resistance, especially in adult cases (Hochberg et al., 2009; Molyneux et al., 2012; Shankland et al., 2012). Lytic induction therapy for EBV-associated malignancies has long been envisioned (Israel and Kenney, 2003; Li et al., 2018; Yiu et al., 2020), but has not yet been realized because few substances, such as ganciclovir, specifically and efficiently kill cells harboring EBV in the lytic cycle. Because METTL3 inhibition can be highly specific and efficient (Figures 4, 6, 8), it is a potential target for anticancer drugs in combination with lytic inducers. To that end, further studies, including *in vivo* preclinical and clinical analyses, are needed.

## Data Availability Statement

The raw data supporting the conclusions of this article will be made available by the authors, without undue reservation.

## Author Contributions

YY and TM: conceptualization, data curation, validation, visualization, and writing—original draft preparation. YY, TW, YS, and TM: methodology. YY, TW, YH, and YS: formal analysis, investigation. TM: resources. TM, TW, YH, YS, and HK: writing—review and editing. TM and HK: supervision and project administration. TM, YS, and HK: funding acquisition. All authors contributed to the article and approved the submitted version.

## Funding

This work was supported by grants-in-aid for Scientific Research from the Ministry of Education, Culture, Sports, Science and Technology (19K07580 to TM 19K22560 and 20H03493 to HK), Japan Agency for Medical Research and Development (JP20wm0325012 to TM and JP21wm0325042 to TM and YS), and the Takeda Science Foundation (to TM).

## Conflict of Interest

The authors declare that the research was conducted in the absence of any commercial or financial relationships that could be construed as a potential conflict of interest.

## Publisher's Note

All claims expressed in this article are solely those of the authors and do not necessarily represent those of their affiliated organizations, or those of the publisher, the editors and the reviewers. Any product that may be evaluated in this article, or claim that may be made by its manufacturer, is not guaranteed or endorsed by the publisher.

## Supplementary Material

Supplementary Figure 1 Importance of METTL3 for efficient production of EBV progeny in HEK293 cells. **(A)** EBV-positive wild-type (Wild) and METTL3-KO (ME3KO) HEK293 cells were transfected with BZLF1 expression plasmids to induce the lytic cycle. Cell proteins were collected before lytic induction [BZLF1(–)] or at 3 days after BZLF1 transfection [BZLF1(+)] and subjected to immunoblotting. IE, E, and L denote immediate-early, early, and late genes, respectively. **(B)** Cell DNA was extracted before lytic induction [BZLF1(–)] or at 3 days after BZLF1 transfection [BZLF1(+)], and EBV DNA was quantified using qPCR. The means ± SDs of three biological replicates normalized to host DNA level are presented. **(C)** Lytic induction was performed as in **(A)**. Culture media were collected on day 4 after transfection, and supernatants were inoculated with EBV-negative Akata cells. The percentage of infected cells was measured based on GFP positivity using FACS. The means ± SDs of three biological replicates are presented. ^****^*p* < 0.001 and ^*****^*p* < 0.0005.

Supplementary Figure 2 Importance of METTL3 for efficient production of EBV progeny in Akata cells. **(A)** EBV-positive wild-type (Wild) and METTL3-KO (ME3KO) Akata cells were treated with anti-IgG to induce the lytic cycle. Proteins were collected before lytic induction [anti-IgG(–)] or at 3 days after anti-IgG addition [anti-IgG(+)] and subjected to immunoblotting. IE, E, and L denote immediate-early, early, and late genes, respectively. **(B)** Cell DNA was extracted before lytic induction [anti-IgG(–)] or at 3 days after anti-IgG addition [anti-IgG(+)], and EBV DNA was quantified using qPCR. The means ± SDs of three biological replicates normalized to host DNA level are presented. **(C)** Lytic induction was performed as in **(A)**. Culture media were collected on day 4, and supernatants were inoculated with EBV-negative Akata cells. The percentage of infected cells was measured based on GFP positivity using FACS. The means ± SDs of three biological replicates are presented. ^***^*p* < 0.005 and ^*****^*p* < 0.0005.
