# Supplementary figures and images for: EBV Exploits RNA m6A Modification to Promote Cell Survival and Progeny Virus Production During Lytic Cycle
Source: Front Microbiol. 2022 Jun 15;13:870816. doi: 10.3389/fmicb.2022.870816 (PMC9240777; doi:10.3389/fmicb.2022.870816)

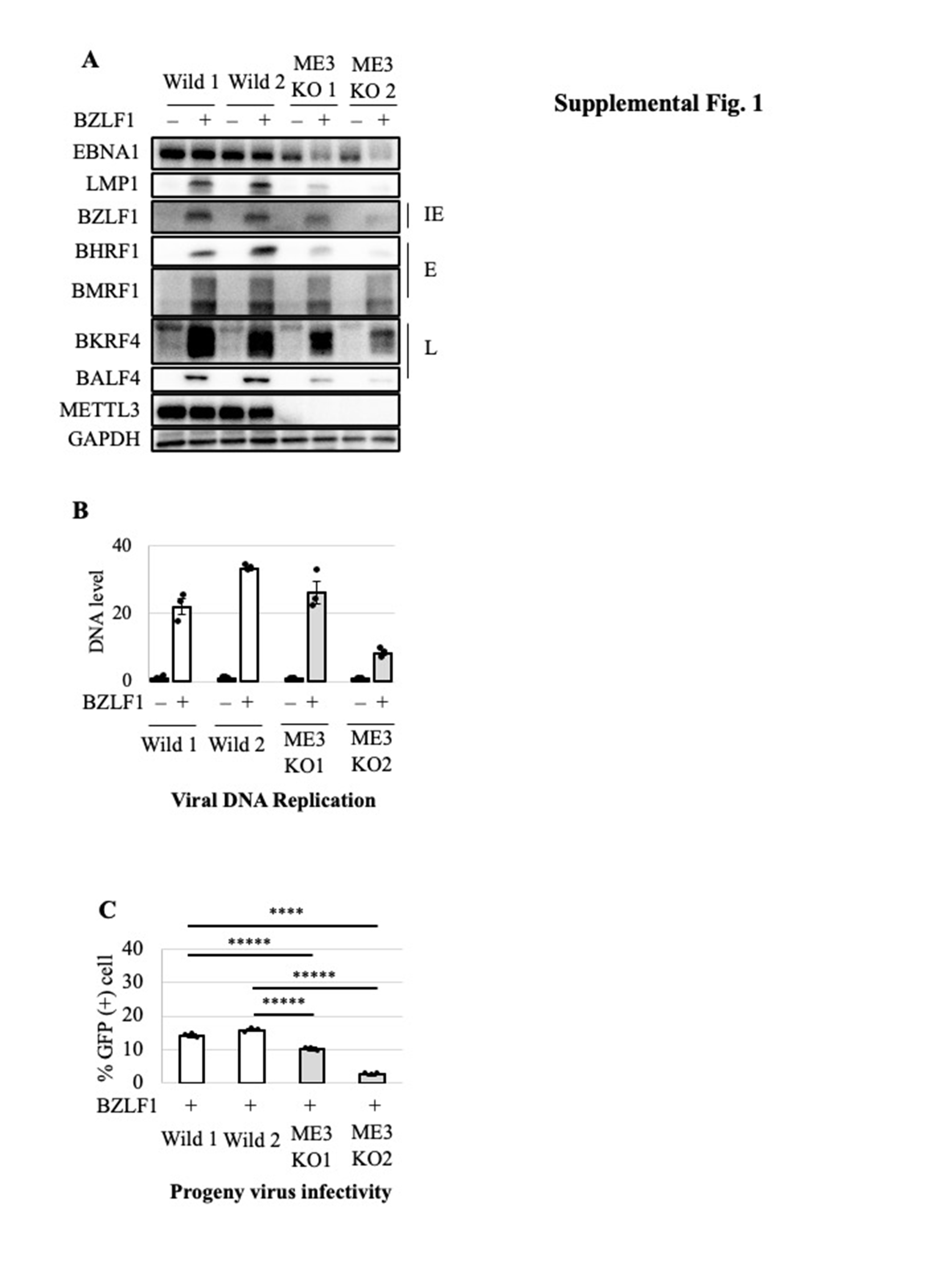

Supplement: Supplementary Figure 1 — Importance of METTL3 for efficient production of EBV progeny in HEK293 cells. (A) EBV-positive wild-type (Wild) and METTL3-KO (ME3KO) HEK293 cells were transfected with BZLF1 expression plasmids to induce the lytic cycle. Cell proteins were collected before lytic induction [BZLF1(–)] or at 3 days after BZLF1 transfection [BZLF1(+)] and subjected to immunoblotting. IE, E, and L denote immediate-early, early, and late genes, respectively. (B) Cell DNA was extracted before lytic induction [BZLF1(–)] or at 3 days after BZLF1 transfection [BZLF1(+)], and EBV DNA was quantified using qPCR. The means ± SDs of three biological replicates normalized to host DNA level are presented. (C) Lytic induction was performed as in (A). Culture media were collected on day 4 after transfection, and supernatants were inoculated with EBV-negative Akata cells. The percentage of infected cells was measured based on GFP positivity using FACS. The means ± SDs of three biological replicates are presented. ****p < 0.001 and *****p < 0.0005. [file Image_1.JPEG]

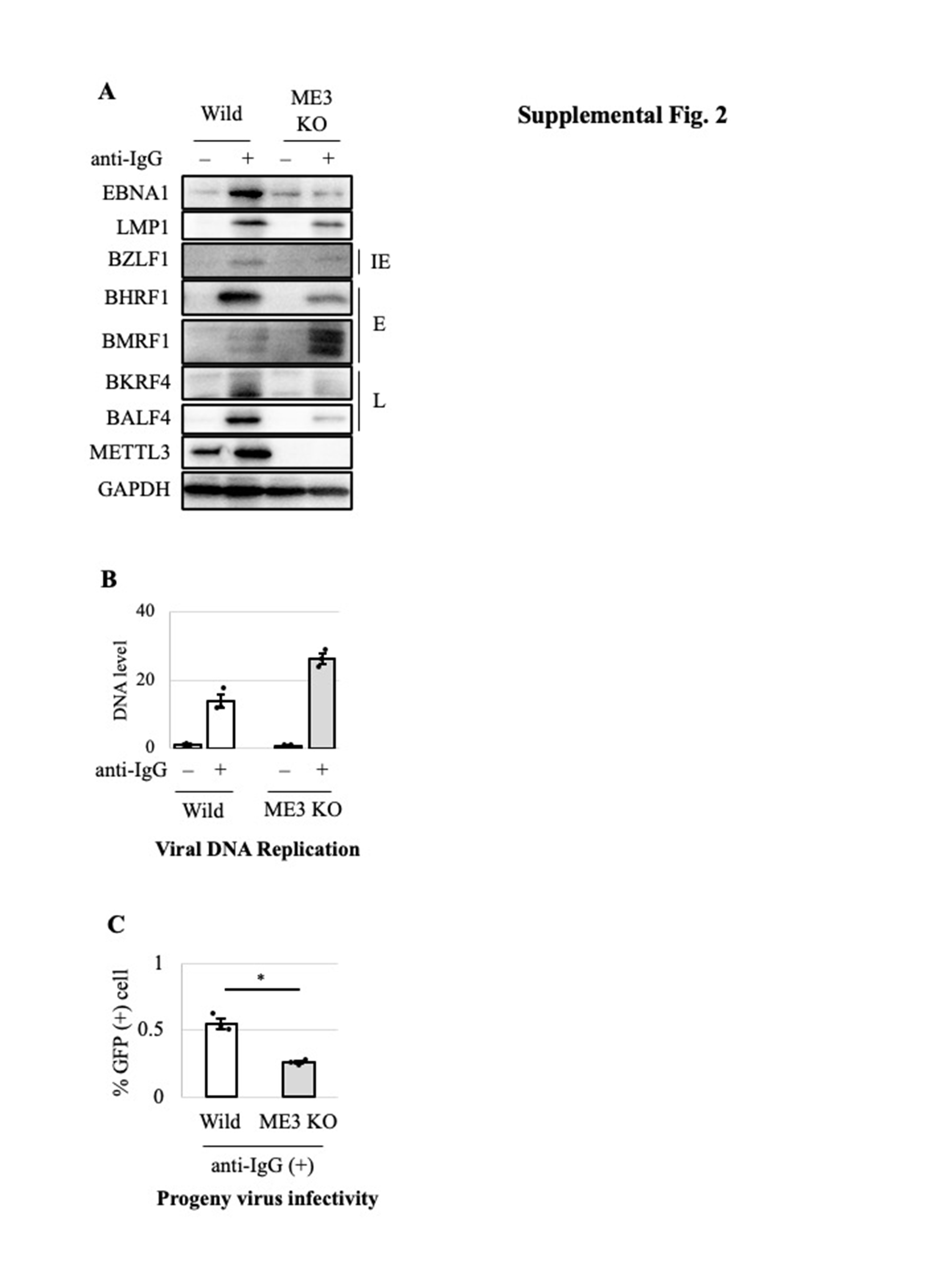

Supplement: Supplementary Figure 2 — Importance of METTL3 for efficient production of EBV progeny in Akata cells. (A) EBV-positive wild-type (Wild) and METTL3-KO (ME3KO) Akata cells were treated with anti-IgG to induce the lytic cycle. Proteins were collected before lytic induction [anti-IgG(–)] or at 3 days after anti-IgG addition [anti-IgG(+)] and subjected to immunoblotting. IE, E, and L denote immediate-early, early, and late genes, respectively. (B) Cell DNA was extracted before lytic induction [anti-IgG(–)] or at 3 days after anti-IgG addition [anti-IgG(+)], and EBV DNA was quantified using qPCR. The means ± SDs of three biological replicates normalized to host DNA level are presented. (C) Lytic induction was performed as in (A). Culture media were collected on day 4, and supernatants were inoculated with EBV-negative Akata cells. The percentage of infected cells was measured based on GFP positivity using FACS. The means ± SDs of three biological replicates are presented. ***p < 0.005 and *****p < 0.0005. [file Image_2.JPEG]
